# Circulating Docosahexaenoic Acid Levels Are Associated with Fetal Insulin Sensitivity

**DOI:** 10.1371/journal.pone.0085054

**Published:** 2014-01-13

**Authors:** Jin-Ping Zhao, Emile Levy, William D. Fraser, Pierre Julien, Edgard Delvin, Alain Montoudis, Schohraya Spahis, Carole Garofalo, Anne Monique Nuyt, Zhong-Cheng Luo

**Affiliations:** 1 Department of Obstetrics and Gynecology, University of Montreal, Montreal, Quebec, Canada; 2 Department of Nutrition, University of Montreal, Montreal, Quebec, Canada; 3 Endocrinology and Nephrology, Laval University Hospital Research Centre, and Department of Medicine, Laval University, Quebec City, Quebec, Canada; 4 Department of Biochemistry, University of Montreal, Montreal, Quebec, Canada; 5 Department of Pediatrics, Sainte-Justine Hospital Research Center, University of Montreal, Montreal, Quebec, Canada; 6 Ministry of Education-Shanghai Key Laboratory of Children's Environmental Health, Xinhua Hospital, Shanghai Jiao-Tong University School of Medicine, Shanghai, China; Virgen Macarena University Hospital, School of Medicine, University of Seville, Spain

## Abstract

**Background:**

Arachidonic acid (AA; C20∶4 n-6) and docosahexaenoic acid (DHA; C22∶6 n-3) are important long-chain polyunsaturated fatty acids (LC-PUFA) in maintaining pancreatic beta-cell structure and function. Newborns of gestational diabetic mothers are more susceptible to the development of type 2 diabetes in adulthood. It is not known whether low circulating AA or DHA is involved in perinatally “programming” this susceptibility. This study aimed to assess whether circulating concentrations of AA, DHA and other fatty acids are associated with fetal insulin sensitivity or beta-cell function, and whether low circulating concentrations of AA or DHA are involved in compromised fetal insulin sensitivity in gestational diabetic pregnancies.

**Methods and Principal Findings:**

In a prospective singleton pregnancy cohort, maternal (32-35 weeks gestation) and cord plasma fatty acids were assessed in relation to surrogate indicators of fetal insulin sensitivity (cord plasma glucose-to-insulin ratio, proinsulin concentration) and beta-cell function (proinsulin-to-insulin ratio) in 108 mother-newborn pairs. Cord plasma DHA levels (in percentage of total fatty acids) were lower comparing newborns of gestational diabetic (n = 24) vs. non-diabetic pregnancies (2.9% vs. 3.5%, *P* = 0.01). Adjusting for gestational age at blood sampling, lower cord plasma DHA levels were associated with lower fetal insulin sensitivity (lower glucose-to-insulin ratio, r = 0.20, *P* = 0.036; higher proinsulin concentration, r = −0.37, *P* <0.0001). The associations remained after adjustment for maternal and newborn characteristics. Cord plasma saturated fatty acids C18∶0 and C20∶0 were negatively correlated with fetal insulin sensitivity, but their levels were not different between gestational diabetic and non-diabetic pregnancies. Cord plasma AA levels were not correlated with fetal insulin sensitivity.

**Conclusion:**

Low circulating DHA levels are associated with compromised fetal insulin sensitivity, and may be involved in perinatally “programming” the susceptibility to type 2 diabetes in the offspring of gestational diabetic mothers.

## Introduction

Gestational diabetes is a common pregnancy complication affecting 3.5% to 7.2% of pregnancies [1]. There remains a lack of effective measures for the prevention of gestational diabetes [2]. Offspring of gestational diabetic mothers are more susceptible to the development of type 2 diabetes in adulthood [3]. Newborns of diabetic mothers tend to have lower cord blood arachidonic acid (AA; C20∶4 n-6) and docosahexaenoic acid (DHA; C22∶6n-3) levels [4], [5]. AA and DHA are vital to the structure and function of cell membranes [6]. In cell culture, long-chain polyunsaturated fatty acids (LC-PUFA) are fundamental to the functional integrity of pancreatic beta-cells [7], [8]. Glucose stimulated insulin release has been shown to be dependent on plasma membrane release of AA in pancreatic islets [9]. Recent animal studies suggest a dose-dependent effect of dietary n-3 LC-PUFA on glucose metabolism and insulin action [10]. A growing body of evidence suggests that fatty acid nutritional status in early life affects fetal tissue lipids as well as neuroendocrine and metabolic pathways relevant to metabolic “programming” [11]. It has been proposed that the low availability of n-3 LC-PUFAs intrauterine may explain the higher incidence of insulin resistance and related disorders in the offspring of diabetic mothers [12]. DHA content in cell membranes and plasma has been associated with insulin sensitivity in postnatal life in humans [13], [14]. In children and adults, levels of plasma and tissue fatty acids have been associated with insulin resistance [13], [15], [16]. It remains unknown whether DHA and AA may affect insulin sensitivity or beta-cell function during fetal life, and whether low circulating DHA or AA levels may be involved in perinatally “programming” the susceptibility to type 2 diabetes in the offspring of gestational diabetic mothers. The primary objective of the present study was to assess whether circulating levels of DHA, AA are associated with insulin sensitivity or beta-cell function in human fetuses/newborns. We also explored whether circulating levels of other fatty acids are associated with fetal insulin sensitivity, and whether there are differences in circulating levels of fatty acids correlated to fetal insulin sensitivity comparing gestational vs. non-diabetic pregnancies. This may offer some clue about whether alterations in circulating levels of certain fatty acids may be involved in impaired fetal insulin sensitivity (“programming”) in gestational diabetic pregnancies.

## Subjects and Methods

### Study design, population and specimen

In a prospective singleton pregnancy cohort study in Montreal, Canada, we have shown that gestational diabetes may impair fetal insulin sensitivity [17]. To further study the potential impact of fatty acids on fetal insulin sensitivity, maternal and cord plasma specimens were used to measure fatty acids in a subset of the cohort. Subjects who reported neither particular diet (e.g. vegetarian) nor taking any supplemental sources of DHA (e.g. fish oil) and with both cord plasma and fasting maternal plasma specimen at 32–35 weeks gestation available for fatty acids assays were eligible for the present study. The final study cohort comprised 108 mother-newborn pairs, including all 24 eligible mothers (and their newborns) with gestational diabetes and a 50% random sample (84 out of 168) of eligible healthy mothers (and their newborns as controls). The original study cohort design and population have been described elsewhere [17]. Briefly, pregnant women without pre-gestational diabetes bearing a singleton fetus without malformations were recruited at 24–28 weeks gestation from three obstetric care centers in Montreal. All women were screened for gestational diabetes at 24–28 weeks gestation by an 1-h 50-g oral glucose tolerance test (OGTT) [17]. If the blood glucose value in 1 h 50 g OGTT was ≥7.8 mmol/L, a diagnostic 75-g 2-h OGTT was carried out. Gestational diabetes was diagnosed if the woman had two of three glucose values exceeding the following cutoffs: fasting 5.3 mmol/L, 1-h 10.0 mmol/L and 2-h 8.6 mmol/L, according to the American Diabetes Association criteria [18]. Upon diagnosis, gestational diabetes was managed by dietary and lifestyle interventions, and by insulin treatment if required, to achieve euglycemia. Venous cord blood specimens were collected *immediately* into EDTA-containing tubes after the delivery of the baby but before the expulsion of the placenta. All collected specimens were kept on ice and stored temporarily in 4°C, and centrifuged at 4°C within 30 minutes after specimen collection. The separated plasma samples were stored in multiple aliquots in −80°C until assays.

### Ethics statement

The study was approved by the research ethics committees of all participating hospitals (Sainte-Justine, Jewish General and Saint Mary’s Hospitals, Montreal, Canada). Written informed consent was obtained from all participants.

### Plasma fatty acids assays

Maternal (32–35 weeks gestation) and cord plasma fatty acids were analyzed by gas chromatography [19]. Briefly, total lipids were extracted from plasma by chloroform/methanol (2∶1) containing 0.01% butylhydroxytoluene [20]. An internal standard, heptadecanoic acid (C17∶0), was added to each sample. The crude lipid extracts were subjected to direct transesterification and then injected into a gas chromatograph using a (90 m×0.32 mm) wall-coated open tubular (WCOT) fused silica capillary column VF-23 ms coated with 0.25 µm thick film (Varian, Canada). Fatty acids (C16–22) were identified by comparison with standards using the same retention times, and the results were expressed as % relative weight (g/100 g of total fatty acids).

### Fetal insulin sensitivity and beta-cell function

Data on surrogate indicators of fetal insulin sensitivity and beta-cell function are available in the original study cohort [17]. Here, we reported their associations with maternal and fetal circulating fatty acids. Cord plasma glucose-to-insulin ratio (mg dl^−1^ µU^−1^ ml^−1^) and proinsulin (pmol/L) level were used as surrogate indicators of fetal insulin sensitivity, and proinsulin-to-insulin ratio as an indicator of fetal beta-cell function [17], [21]. Plasma glucose (mmol/L, 1 mmol/L = 18 mg/dl) was measured by the glucose oxidase method, plasma insulin (μU/ml, 1 pmol/ =  6 µU/mL) by a chemiluminescent immunometric assay, and plasma proinsulin by a quantitative ELISA kit, with intra-assay and inter-assay coefficients of variation in the range of 2–6% [17].

### Statistical analysis

Plasma fatty acids levels were expressed as mean ± SD. Biomarker data were assessed for Normality in distribution using the Kolmogorov-Smirnov test. Chi-square tests were applied to test for differences in proportions for categorical variables, and t-tests for differences in means for continuous variables with normal data distribution, and Wilcoxon tests for differences in continuous variables with skewed data distribution. Log transformation was applied for variables with skewed crude data distribution to normalize data distribution in correlation and regression analyses. Partial correlation was used to assess the associations of fatty acids levels with indicators of fetal insulin sensitivity and beta-cell function adjusting for gestational age at blood sampling. Generalized linear models were used to assess the differences in fatty acids levels between gestational diabetic and non-diabetic pregnancies, and the changes in fetal insulin sensitivity and beta-cell function indicators per SD increase in each fatty acid adjusting for maternal and newborn characteristics, including maternal ethnicity (French mother tongue - the majority group in Quebec, others), parity (primiparous: yes/no), pre-pregnancy body mass index (BMI; kg/m^2^, per SD increase), smoking (yes/no) and alcohol use (yes/no), infant sex, gestational age (weeks) and mode of delivery (caesarean section: yes/no). All data management and analyses were conducted using Statistical Analysis System (SAS), version 9.0 (SAS Institute, Cary, North Carolina, USA). Two-sided *P* values <0.05 were considered statistically significant. The study had a power of 75% to detect an absolute correlation coefficient of 0.3 or stronger association in testing the primary hypothesis that cord plasma AA and DHA concentrations are associated with fetal insulin sensitivity or beta-cell function accounting for multiple tests.

## Results

### Participant characteristics

Women with gestational diabetes had significantly higher pre-pregnancy body mass index, and were more likely to have a caesarean section delivery than non-diabetic women (**Table 1**). Cord plasma insulin and proinsulin concentrations were significantly higher while glucose-to-insulin ratios significantly lower comparing the newborns of gestational diabetic vs. non-diabetic mothers. There was no significant difference in gestational age at delivery, cord blood glucose concentration or proinsulin-to-insulin ratio between the two groups.

**Table 1:** Maternal and neonatal characteristics of study participants.

	Control (n = 84)	GDM (n = 24)	P Value*
**Mothers**			
**Ethnicity - French Canadians**	44 (52.4%)	13 (54.2%)	0.13
**Age (years)**	31.3±5.4	32.3±3.6	0.44
**Primiparous**	28 (33.3%)	7 (29.2%)	0.70
**Pre-pregnancy BMI (kg/m^2^)**	23.8±4.9	27.0±6.7	0.01
**Smoking**	6 (7.1%)	1 (4.2%)	0.60
**Alcohol use**	12 (14.3%)	6 (25.0%)	0.21
**Newborns**			
**Caesarean section delivery**	23 (27.4%)	15 (62.5%)	<0.0001
**Gestational age (week)**	38.8±1.6	38.2±2.0	0.12
**Cord plasma**			
**Glucose (mmol/l)**	4.8±1.0	4.6±0.8	0.47
**Insulin (pmol/l)**	28.7±20.2	54.6±30.9	<0.0001
**Proinsulin (pmol/l)**	15.7±11.4	27.8±17.0	<0.0001
**Glucose (mg/dl)-to-insulin (µU/ml) ratio**	25.0 28.7±20.7	11.2 12.3±7.5	<0.0001
**Proinsulin-to-insulin ratio**	0.59 0.7±0.5	0.49 0.6±0.4	0.34

Data presented are means ± SD, or n (%). GDM = gestational diabetes mellitus.

*P* values in Chi-square tests for differences in proportions for categorical variables, and t-tests for differences in means for continuous variables with normal data distribution, and Wilcoxon tests for differences in continuous variables with skewed data distribution.

### Maternal and cord plasma fatty acids levels

Cord plasma DHA (but not AA) levels were significantly lower in the newborns of gestational diabetic pregnancies than non-diabetic pregnancies (∼ 17.1% lower in relative weight, *P* = 0.01) (**Table 2**), despite similar cord blood glucose concentrations (4.6 vs. 4.8 mmol/L, *P* = 0.47) indicating adequate management of diabetes in the study cohort [17]. A similar pattern was observed for eicosapentaenoic acid (EPA; C20∶5n-3; ∼33.3% lower in gestational diabetic pregnancies, *P* = 0.02). These differences in cord plasma DHA and EPA between gestational diabetic and non-diabetic pregnancies remained significant or marginally significant after adjusting for maternal (ethnicity, parity, pre-pregnancy BMI, smoking and alcohol use) and newborn (sex, gestational age and mode of delivery) characteristics (adjusted *P* = 0.03 for EPA; adjusted *P* = 0.08 for DHA). In addition, compared to newborns of non-diabetic pregnancies, newborns of gestational diabetic pregnancies had significantly higher total monounsaturated fatty acid (∑MUFA, ∼4.5% higher, *P* = 0.03), but significantly lower n-3 LC-PUFA (∼20.5% lower, *P* = 0.04) and n-3/n-6 ratio (*P* = 0.05, Table 2). These differences remained significant or marginally significant after adjusting for maternal and newborn characteristics (adjusted *P* = 0.06 for ∑MUFA; adjusted *P* = 0.03 for n-3 LC-PUFA; adjusted *P* = 0.04 for n-3/n-6 ratio). There were no significant differences in the cord plasma levels of AA, arachidic (C20∶0), and α-linolenic acid (C18∶3n-3) between newborns of gestational diabetic and non-diabetic pregnancies. There were no significant differences in any maternal plasma fatty acids levels at 32-35 weeks gestation between gestational diabetic and non-diabetic pregnancies (Table S1).

**Table 2:** Cord plasma fatty acids in newborns of mothers with versus without gestational diabetes mellitus.

	Weight (% of total fatty acids)			
	Control (n = 84)	GDM (n = 24)	Crude *P*	Adjusted *P* *
16∶0	28.6±3.2	29.4±1.9	0.28	0.13
18∶0	10.2±1.8	10.3±2.0	0.91	0.99
20∶0	0.5±0.1	0.5±0.1	0.97	0.74
∑ SFA	43.5±3.4	44.7±2.8	0.10	**0.05**
16∶1n-7	3.5±0.8	3.6±0.7	0.45	0.48
18∶1n-9	15.5±2.0	15.9±1.5	0.31	0.27
∑MUFA	24.5±2.2	25.6±2.2	**0.03**	**0.06**
18∶2	10.1±1.9	9.9±1.9	0.69	0.76
20∶3	2.6±0.7	2.5±0.5	0.38	0.28
20∶4 (AA)	11.7±2.0	11.3±1.6	0.35	0.83
22∶4	0.6±1.9	0.4±0.1	0.60	0.28
∑n-6 PUFA	25.8±2.5	24.7±2.5	0.06	0.07
18∶3	0.2±0.2	0.2±0.1	0.55	0.60
20∶5	0.3±0.1	0.2±0.1	**0.02**	**0.03**
22∶5	0.4 ± 1.9	0.2±0.1	0.54	0.24
22∶6 (DHA)	3.5±1.1	2.9±0.8	**0.01**	**0.08**
∑n-3 PUFA	4.4±2.1	3.5±0.9	**0.04**	**0.03**
∑n-3/∑ n-6	0.17±0.07	0.14±0.04	**0.05**	**0.046**

Data presented are means ± SD. GDM = gestational diabetes mellitus; SFA = saturated fatty acid; MUFA = mono-unsaturated fatty acid; PUFA = polyunsaturated fatty acid; AA =  Arachidonic acid; DHA = docosahexaenoic acid.

*P* values adjusted for infant sex, gestational age, mode of delivery, maternal ethnicity, parity, pre-pregnancy BMI, smoking and alcohol use.

P values in **bold** indicate significant differences (*P* <0.05) comparing gestational diabetic vs. non-diabetic (control) pregnancies.

### Correlations in plasma fatty acids between mothers and neonates

Plasma fatty acid levels in maternal versus cord blood were positively correlated for DHA, EPA and docosapentaenoic acid (C22∶5n-3), stearic (C18∶0), C20∶0 and dihomo-gamma-linolenic acid (C20∶3n-6) (partial r: 0.27 to 0.58, all *P*<0.01, **Table 3**).

**Table 3:** Partial correlations between maternal and fetal/cord plasma fatty acids and between cord plasma fatty acids and indicators of fetal insulin sensitivity and beta-cell function*.

Cord plasma	Maternal plasma	Cord plasma					
fatty acids	fatty acids	Glucose/insulin		Proinsulin	Proinsulin/insulin		
16∶0	0.18	0.02		−0.02			0.05
18∶0	**0.58^c^**	**−0.20^a^**	**0.28^b^**			0.02	
20∶0	**0.40^c^**	**−0.38^c^**	**0.39^c^**			−0.09	
∑ SFA	0.15	−0.15	**0.22^a^**			0.06	
16∶1n-7	0.14	0.03	−0.05			0.07	
18∶1n-9	−0.008	0.02	−0.11			−0.11	
∑ MUFA	0.06	−0.08	0.07			−0.007	
18∶2	0.14	0.19	**−0.25** ^b^			−0.01	
20∶3	**0.28^b^**	**−0.21^a^**	0.16			−0.11	
20∶4 (AA)	0.17	−0.03	−0.08			−0.08	
22∶4	0.09	0.11	−0.02			0.07	
∑ n-6 PUFA	0.14	0.17	**−0.23** ^a^			−0.04	
18∶3	0.02	**0.30^b^**	**−0.20^a^**			0.10	
20∶5	**0.53^c^**	0.06	−0.15			−0.08	
22∶5	**0.27^b^**	0.12	−0.03			0.07	
22∶6 (DHA)	**0.47^c^**	**0.20^a^**	**−0.37^c^**			−0.09	
∑ n–3 PUFA	**0.40^c^**	**0.24^a^**	**−0.24^a^**			0.03	
∑ n–3/∑ n-6	**0.55^c^**	**0.22^a^**	**−0.23^a^**			0.02	

SFA = saturated fatty acid; MUFA = mono-unsaturated fatty acid; PUFA = polyunsaturated fatty acid; AA =  Arachidonic acid; DHA = docosahexaenoic acid.

Partial correlation coefficients adjusted for gestational age at blood sampling.

Correlation coefficients in **bold**, ^a^ *P*<0.05, ^b^ *P* <0.01, ^c^ *P* <0.001.

### Correlations between cord plasma fatty acids and fetal metabolic parameters

**Figure 1:**
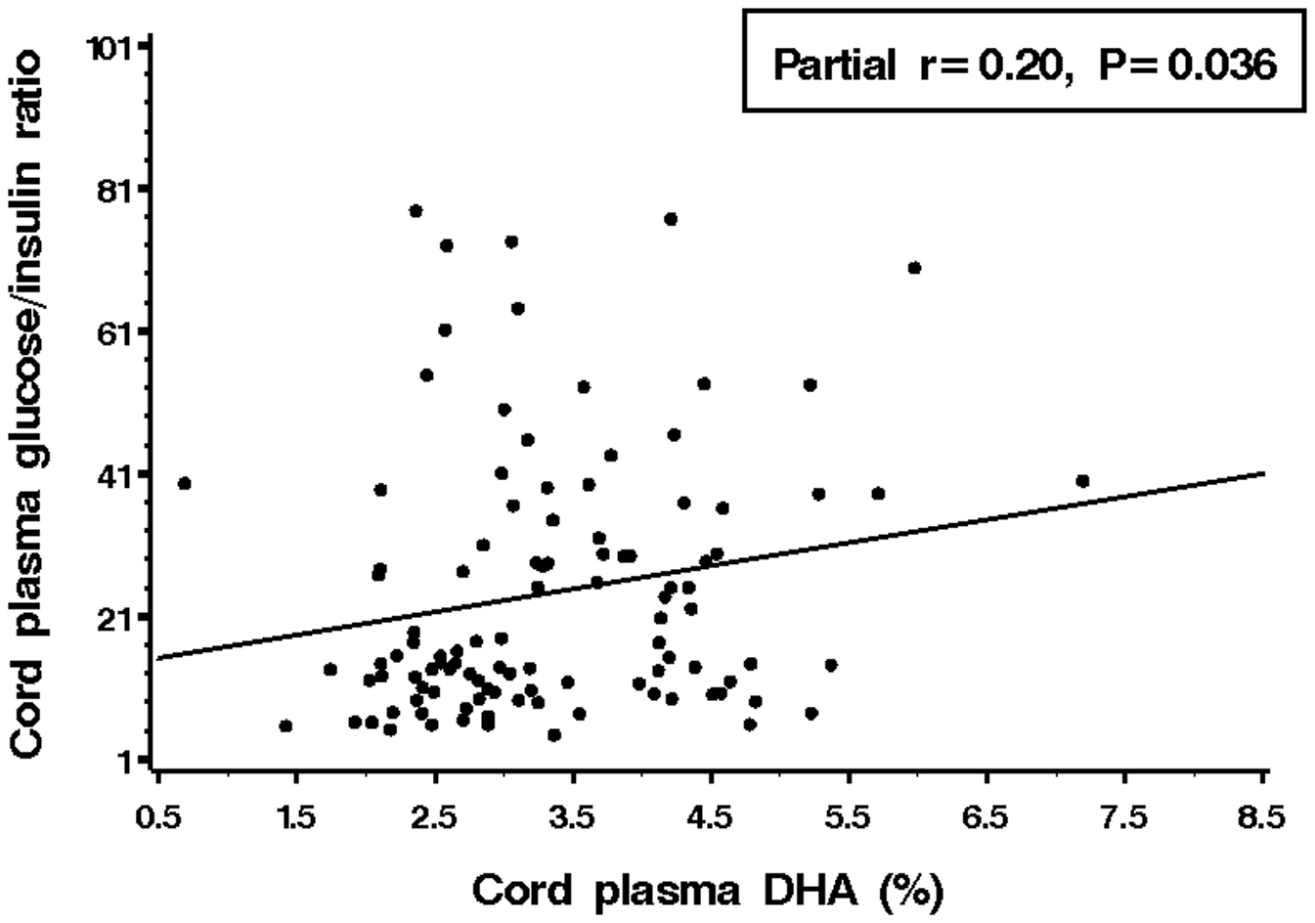
Positive correlation between cord plasma DHA and glucose-to-insulin ratio.

Partial correlation analyses adjusting for gestational age at blood sampling revealed that cord plasma glucose-to-insulin ratio (an indicator of fetal insulin sensitivity) [17], [21] was positively correlated with cord plasma DHA (partial r = 0.20, *P* = 0.036, **Figure 1**) and its essential fatty acid metabolic precursor α-linolenic acid (partial r = 0.30, *P* = 0.002), as well as n-3 LC-PUFA (partial r = 0.24, *P* = 0.01) and n-3/n-6 ratio (partial r = 0.22, *P* = 0.02) (**Table 3**). In contrast, saturated fatty acids C18∶0 (partial r = −0.20, *P* = 0.04) and C20∶0 (partial r = −0.38, *P*<0.0001), as well as n-6 LC-PUFA C20∶3n-6 (partial r = -0.21, *P* = 0.03) were all negatively correlated with cord plasma glucose-to-insulin ratio.

**Figure 2:**
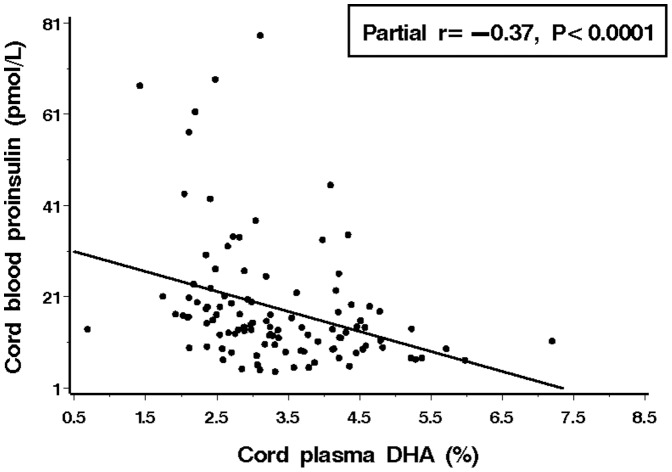
Negative correlation between cord plasma DHA and proinsulin concentration.

Cord plasma DHA (partial r = −0.37, *P*<0.0001, **Figure 2**), α-linolenic acid (partial r = −0.20, *P* = 0.04), n-3 LC-PUFA (partial r = −0.24, *P* = 0.01) and n-3/n-6 ratio (partial r = −0.23, *P* = 0.02) were all negatively correlated with cord plasma proinsulin concentration (indicating a positive correlation to insulin sensitivity) (**Table 3**). Similarly, cord plasma C18∶2n-6 (partial r = −0.25, *P* = 0.01) and n-6 LC-PUFA (partial r = −0.23, *P* = 0.02) were negatively correlated with cord plasma proinsulin concentration. In contrast, saturated fatty acids C18∶0 (partial r = 0.28, *P* = 0.004) and C20∶0 (partial r = 0.39, *P*<0.0001) and total saturated fatty acids (partial r = 0.22, *P* = 0.02) were all positively correlated with cord plasma proinsulin concentration. There was no significant correlation in cord plasma AA, EPA, docosapentaenoic acid or monounsaturated fatty acid concentration with cord plasma glucose-to-insulin ratio, proinsulin concentration or proinsulin-to-insulin ratio (all *P*>0.05).

There was no significant correlation between any cord plasma fatty acid and proinsulin-to-insulin ratio (an indicator of fetal beta-cell function), or between any maternal plasma fatty acid and cord plasma indicators of fetal insulin sensitivity or beta-cell function (data not shown).

### Adjusted change in insulin sensitivity per SD increase in cord plasma fatty acids

Adjusting for gestational diabetes, maternal ethnicity, parity, pre-pregnancy body mass index, smoking and alcohol use, infant sex, gestational age and mode of delivery, cord plasma fatty acid C20∶0, C20∶3n-6, DHA and α-linolenic acid remained significantly associated with cord plasma glucose-to-insulin ratio and proinsulin concentration. Each SD increase in C20∶0 and C20∶3n-6 was associated with a significant decrease in glucose-to-insulin ratio by 6.1 (95% CI: 2.1 to 10.1, *P* = 0.003) and 4.2 (95% CI: 0.7 to 7.8, *P* = 0.02), respectively. Each SD increase in C20∶0 was associated with a significant increase in fetal plasma proinsulin by 4.2 (95% CI: 1.6 to 6.8, *P* = 0.002) pmol/L. Each SD increase in DHA was associated with a significant decrease in cord plasma proinsulin by 3.4 (95% CI: 1.0 to 5.9, *P* = 0.007) pmol/L, while each SD increase in α-linolenic acid was associated with a decrease in cord plasma proinsulin by 2.5 (95% CI: 0.1 to 4.9, *P* = 0.04) pmol/L.

## Discussion

To our knowledge, this is the first human study on the associations between circulating levels of fatty acids and fetal insulin sensitivity. We observed positive correlations of fetal insulin sensitivity with cord plasma DHA, α-linolenic acid, n-3 LC-PUFA and n-3/n-6 LC-PUFA ratio, while cord plasma n-6 LC-PUFA C20∶3n-6 and saturated fatty acids C18∶0 and C20∶0 were negatively correlated with fetal insulin sensitivity. However, among these fatty acids associated with surrogate biomarkers of fetal insulin sensitivity, only DHA and n-3/n-6 LC-PUFA ratio were significantly different between gestational diabetic and non-diabetic pregnancies.

LC-PUFA are a fundamental component of cell membranes [6] where they modulate functional properties such as fluidity, permeability for metabolite exchange, activity of membrane-bound enzymes and receptors, and electrical and humoral signal transduction [6]. The insulin receptor is embedded in the lipid bilayer of the plasma membrane and is sensitive to the surrounding lipid environment [22]. Cells from animals fed on a high saturated fatty acid diet bound significantly less insulin than did cells from animals fed on a high LC-PUFA diet [22]. Feeding neonatal pigs with a n-3 LC-PUFA rich diet increased insulin sensitivity through enhancing insulin signalling [22]. Epidemiologic studies have shown an inverse relation between dietary n-3 fatty acids intake and the incidence of type 2 diabetes [23]. Previous clinical studies have reported the relationships between plasma or tissue fatty acids and insulin resistance and related disorders in adults [15], [24], youth [13], [25] and infants [14]. Higher n-3 LC-PUFA levels have been associated with improved insulin action, while higher saturated fatty acids levels have been associated with insulin resistance [13]-[15], [24], [25]. The present study adds important new data on the association between circulating fatty acids levels and fetal insulin sensitivity. Addressing this issue in early life is important as the perinatal period represents a critical “programming” window and an opportunity for early life preventive interventions. We showed that higher cord plasma DHA concentration and higher n-3/n-6 LC-PUFA ratio were associated with better fetal insulin sensitivity, while cord plasma saturated fatty acids C18∶0 and C20∶0 were negatively correlated with fetal insulin sensitivity, suggesting a positive impact of certain n-3 fatty acids and a negative impact of saturated fatty acids on fetal insulin sensitivity. This is plausible as LC-PUFAs are not only important structural elements of cell membranes, but together with their eicosanoid products are implicated in modulating gene expression [11].

Our data confirmed that newborns of gestational diabetic pregnancies have lower cord plasma DHA levels [4], [5], [12]. We observed lower cord plasma concentrations of DHA and n-3 LC-PUFA, but no significant difference in AA comparing the newborns of gestational diabetic versus non-diabetic pregnancies. The low cord blood DHA and AA in diabetic pregnancies may be a reflection of low maternal status in combination with placental sequestration [26] and increased fetal utilization [27]. The lack of difference in maternal circulating fatty acids levels indicates that the lower fetal circulating DHA levels in gestational diabetic pregnancies may be largely a problem of compromised maternal-placental-fetal DHA transportation, or impaired fetal DHA synthesis. DHA can be obtained directly from the diet or can be endogenously synthesized from α-linolenic acid in the liver [28]. The biosynthesis of DHA requires Δ-6 and Δ-5 desaturase that are insulin dependent [29]. Faas and Carter found a 48% inhibition of Δ6 desaturase activity in the liver microsomes of type 1 diabetic rats [30]. There is a lack of data concerning whether the activities of Δ-6 and Δ-5 desaturase are affected in gestational diabetes. However, it is plausible that this function could be depressed as seen in similar insulin resistance conditions such as type 2 diabetes and obesity [31]. In addition, female hormone estrogen had significant effect on Δ-6 and Δ-5 desaturase gene expression [32]. Significantly higher plasma and liver phospholipids DHA have been observed in female than male rats accompanied by greater gene expression and activity of Δ6- and Δ5- desaturases [32]. Indeed, women of reproductive age have greater capacity in the conversion of α-linolenic acid to DHA than men [33]. A recent population-based study confirmed the higher concentrations of EPA and DHA and greater α-linolenic acid to DHA conversion in women than in men, regardless of dietary n-3 LC-PUFA intake [34]. Similarly, pregnancy is associated with increased concentrations of DHA in plasma and tissues [33], as plasma levels of estrogen increased sharply during gestation [35]. This may occur by the establishment of DHA reserves in adipose tissue which can be mobilized during pregnancy, and/or by enhanced ability of DHA synthesis from its essential fatty acid precursor α-linolenic acid [36]. Low estrogen levels have been observed in type 1 and gestational diabetic pregnancies [35], which may have affected the normal up-regulation of Δ5 and Δ6 desaturase in pregnancy.

The insulin resistance syndrome has become highly prevalent in Canadian youth in recent decades [37]. Up to 30% of youth with type 2 diabetes may have been exposed to maternal diabetes *in utero* [38]. We have demonstrated that gestational diabetes may impair fetal insulin sensitivity and thus “program” the susceptibility to type 2 diabetes [17]; this finding has been validated recently in an independent cohort [39]. It remains unknown whether any nutrients may be involved in “programming” this susceptibility. We observed that lower circulating DHA, higher C18∶0 and C20∶0 saturated fatty acids levels were associated with compromised fetal insulin sensitivity. However, only fetal circulating DHA levels were significantly different between diabetic and non-diabetic pregnancies. A strong positive correlation was observed between maternal and fetal DHA levels, indicating the importance of maternal DHA status for the fetus. Several animal studies have observed some beneficial impact of maternal dietary intervention in modifying the susceptibility of offspring to metabolic disorders [11]. In Western diets, the intake of α-linolenic acid is about 10 to 20-fold greater than that of DHA [40], [41]. For example, the mean intake as estimated by a food-frequency questionnaire at 28-35 weeks gestation was 1.6 g/d for α-linolenic acid and 0.16 g/d for DHA in pregnant women living in Vancouver, Canada [42], indicating a need for DHA supplementation in pregnancy.

The main strength of the current study is the relatively large prospective cohort with robust data on the relationships between cord plasma fatty acids and fetal metabolic parameters. The main limitation is the observational nature of the study. We could not differentiate whether the low cord plasma DHA levels observed in gestational diabetes are a cause or consequence of impaired fetal insulin sensitivity in gestational diabetes. However, considering the well-established positive association between DHA and insulin sensitivity in children [13], [14], a positive impact of DHA on fetal insulin sensitivity is a plausible data interpretation. Another limitation is that we could not obtain fasting cord blood specimen as this is practically difficult at birth. The newborns were not in a uniform metabolic state. However, this would only tend to increase noise variations and bias the associations towards the null.

In conclusion, low circulating fetal DHA levels are associated with compromised fetal insulin sensitivity, and may be involved in “programming” the susceptibility to type 2 diabetes in the offspring of gestational diabetic women. The findings suggest a potential opportunity of early life nutritional interventions (e.g. DHA supplementation) to halt adverse metabolic programming to decrease the incidence of type 2 diabetes in future generations.

## Supporting Information

Table S1 **Maternal plasma fatty acids at 32-35 weeks gestation.**
(DOCX)
