# Supplementary material for: Circulating Docosahexaenoic Acid Levels Are Associated with Fetal Insulin Sensitivity
Source: PLoS One. 2014 Jan 13;9(1):e85054. doi: 10.1371/journal.pone.0085054 (PMC3890289; doi:10.1371/journal.pone.0085054)
Supplement: Table S1 — Maternal plasma fatty acids at 32-35 weeks gestation. (DOCX) [file pone.0085054.s001.docx]

**Table S1.** Maternal plasma fatty acids at 32-35 weeks gestation

|  | Weight (% of | total fatty acids) |  |
| --- | --- | --- | --- |
|  | Control (n=84) | GDM (n=24) | *P** |
| 16:0 | 28.6 ± 2.6 | 29.0 ± 2.2 | 0.52 |
| 18:0 | 5.1 ± 1.0 | 5.1 ± 0.6 | 0.69 |
| 20:0 | 0.20 ± 0.04 | 0.18 ± 0.04 | 0.14 |
| ∑ SFA | 37.5 ± 3.0 | 37.4 ± 2.3 | 0.91 |
| 16:1n-7 | 2.1 ± 0.8 | 1.7 ± 0.5 | 0.05 |
| 18:1n-9 | 20.4 ± 2.3 | 21.2 ± 2.1 | 0.14 |
| ∑MUFA | 25.3 ± 2.6 | 26.0 ± 2.3 | 0.27 |
| 18:2 | 26.8 ± 3.5 | 26.4 ± 2.4 | 0.61 |
| 20:3 | 1.3 ± 0.3 | 1.3 ± 0.3 | 0.67 |
| 20:4 (AA) | 4.7 ± 1.0 | 4.9 ± 1.1 | 0.29 |
| 22:4 | 0.14 ± 0.05 | 0.13 ± 0.04 | 0.62 |
| ∑n-6 PUFA | 33.4 ± 3.9 | 33.1 ± 3.0 | 0.77 |
| 18:3 | 0.76 ± 0.2 | 0.74 ± 0.2 | 0.57 |
| 20:5 | 0.27 ± 0.2 | 0.22 ± 0.1 | 0.16 |
| 22:5 | 0.22 ± 0.06 | 0.19 ± 0.06 | 0.07 |
| 22:6 (DHA) | 1.72 ± 0.4 | 1.66 ± 0.5 | 0.57 |
| ∑n-3 PUFA | 3.0 ± 0.6 | 2.8 ± 0.7 | 0.24 |
| ∑n-3/∑ n-6 | 0.091 ± 0.02 | 0.086 ± 0.02 | 0.24 |

Data presented are means ± SD. GDM=gestational diabetes mellitus.

**P* values comparing gestational diabetic vs. non-diabetic pregnancies.
